# Prognostic prediction tools and clinician communication: a qualitative study of the effect of the STUMBL tool on clinical practice

**DOI:** 10.1186/s12873-020-00331-0

**Published:** 2020-05-11

**Authors:** Claire O’Neill, Hayley A. Hutchings, Zoe Abbott, Ceri Battle

**Affiliations:** 1 Institute of Life Science 2, Swansea University Medical School, Swansea University, Singleton Park, Swansea, SA2 8PP UK; 2 Division of Population Medicine, Cardiff University, Fifth Floor, Neuadd Meirionnydd, Heath Park, Cardiff, CF14 4YS UK; 3 Morriston Hospital, Abertawe Bro Morgannwg University Health Board, Heol Maes Eglwys, Swansea, SA6 6NL UK; 4 Swansea University Medical School, Swansea University, Singleton Park, Swansea, SA2 8PP UK

## Abstract

**Background:**

In recent years, researchers and clinicians have been developing prognostic prediction tools (PPTs) as a way of identifying patients at risk of deterioration. The use of PPTs in the clinical environment not only impacts the risk of adverse outcomes for patients, but the use of these tools also effect clinical practice. Much attention has been paid to the clinical performance of PPTs. But more insight is needed on how the use of PPTs impacts clinical practice. The objective of this study was to map some of the ways in which PPTs effect clinical practice.

The STUMBL (STUdy evaluating the impact of a prognostic model for Management of BLunt chest wall trauma patients) feasibility trial evaluated the use of a new prognostic prediction tool (PPT) to guide the management blunt chest wall trauma patients in the emergency departments (ED). The trial was undertaken between October 2016 and September 2018 and conducted at four sites in England and Wales. Nested within the feasibility trial was a qualitative study aimed at understanding how ED clinicians experienced and used the PPT. The qualitative methods included a focus group and telephone interviews with 9 ED clinicians. This study focused on participant perceptions of the feasibility and use of the STUMBL tool on clinical practice in the ED.

**Results:**

Clinical practice is reshaped as a result of the introduction of the STUMBL PPT into the clinical environment. The PPT enhanced reflexive awareness of prognostic practice; facilitated communication between patients and professionals; helps to guide patient outcomes; and provides a common ground for clinician discussion on prognostication.

**Conclusions:**

The qualitative data collected offered useful insights into the ways in which the tool changes clinical practice. This was a small study of the effect of one kind of PPT on clinical practice. Nevertheless, this study maps areas in which clinical practice is affected by the introduction of a PPT into the clinical environment. More research is needed to better understand these effects, and to understand how these tools become embedded in clinical practice over the longer term.

## Background

While traditionally one of the three constitutive branches of clinical activity, prognostication is a complex and challenging area [1]. As modern medicine developed increasingly accurate diagnoses which also provided indications of the likely outcome of a disease, prognosis was subsumed into diagnosis leading to an ellipsis of prognostic thinking within modern medicine [2]. Nevertheless, prognostication remains a central concern in end of life and critical care. In both areas, prognostication presents challenges for clinicians and patients interested in planning care and making decisions about medical interventions [3, 4].

A number of concerns may deter clinicians from prognosticating. These may include a fear that a prognosis becomes a self-fulfilling prophesy [5]; that it may jeopardise the clinician patient relationship [6]; or clinicians may lack confidence in the prognosis provided [7]. To address these challenges, researchers have been turning attention to the potential of prognostic prediction tools (PPTs) that “may provide the evidence-based input for shared decision-making, by providing estimates of the individual probabilities of risks and benefits” [8]. While a number of studies have focused on the effectiveness and usefulness of PPTs [9] and on the impact of these tools on clinician confidence [3], less attention has been paid to the usefulness of such interventions in clinical practice [10]. Nevertheless, the integration of PPTs into medical care will rely on the provision of tools that clinicians not only find accurate, but also useful.

In this paper we analyse data collected as part of a study on the feasibility of utilising a PPT in a critical care environment. The STUMBL (STUdy evaluating the impact of a prognostic model for Management of BLunt chest wall trauma patients) study was a multi-site feasibility trial set up to understand the feasibility of using a PPT in an emergency department (ED) setting with patients presenting with blunt chest wall trauma [11]. The tool takes five risk factors into account [12]. These include the patient’s age, number of rib fractures, pre-existing chronic lung disease, use of preinjury anticoagulants and oxygen saturation on initial assessment in the ED. The patient is scored on each risk factor in the tool and the total score is used to guide the clinician in the ED as to whether the patient should be admitted as an in-patient (Intensive Care Unit/High Dependency Unit or ward) or can be safely discharged home. Scoring of this PPT is simple and is intended to be calculated manually at the patient bedside.

Overall the PPT developed for the STUMBL trial showed that risk to patients can be accurately stratified by taking together demographic and clinical variables that are routinely collected on initial assessment of the blunt chest-wall trauma patient in the ED. The objective of the feasibility study was to explore the acceptability and performance of the tool. The tool was found to perform safely and effectively in the ED [12]. Alongside the feasibility study, we conducted qualitative research on the experience of clinicians incorporating this tool into their clinical activity. In this paper we explore the experiences of clinicians by building on Baker and Gerdin’s [10] analysis of the process of building a good prognostic tool. Our goal is to illuminate the role played by the PPTs for clinicians, and to inform the development of future studies on the implementation of PPTs in clinical practice.

In their review of prognostic prediction models for critical care, Baker and Gerdin [10] distinguish four characteristics they associate with a useful model. (1) If a prediction tool is to be useful, it must demonstrate good (but not perfect) predictive performance. (2) A model should also be “quick, easy, user-friendly and acceptable to health workers” [10]. (3) A model should provide a clinician with assistance in difficult situations. From this it follows that (4) that a prediction model guides action by indicating what should be done. Finally, (5) a good model will improve care by leading to improved outcomes for patients. In this paper, we focus on steps 2, 3 and 4. We do not address the issue of predictive performance (step 1) here. The model used here has been demonstrated to have excellent discrimination. This trial was only embarked on following this earlier validation work which has shown that clinicians can confidently assess whether the patient with the higher risk prediction using the model will develop complications following blunt chest-wall trauma, compared to the patients with low risk predictions who will not develop complications [11, 12]. Nor do we assess the effect of this PPT on patient outcomes (step 5), as this is beyond the remit of the feasibility study. Baker and Gerdin, highlight that the existing literature on prognostic prediction models has tended to focus on performance and make the case that “the implementation methods themselves should be studied to better understand how such models can best be introduced into hospitals” [10]. Baker and Gerdin say little about how PPTs work, what insights may be unlocked for clinicians using PPTs, and how these could be captured.

This paper unpacks the three middle elements of a good prognostic model as set out in Baker and Gerdin [10]. Their model would benefit from a closer investigation of how PPTs enable clinicians to hold inter and intra-personal conversations about how to manage the patient.

## Methods

STUMBL was a multi-site step wedged feasibility study of the acceptability of using a prognostic model to guide decision making on blunt chest wall trauma in the ED. A total of 177 patients were consented into the study at four sites – the Royal Gwent Hospital, Musgrove Park Hospital, Salford Royal Hospital and the Manchester Royal Infirmary [11]. The study was undertaken between October 2016 to September 2018, with the qualitative component conducted in the months of February, March and April of 2018. The qualitative work was undertaken with the clinical and research teams involved in the feasibility study to understand how the prognostic tool was adopted and used by clinicians as part of their diagnosis and treatment of this patient group. The results of the feasibility trial are reported elsewhere [12].

The qualitative researcher attended the study training sessions and gathered feedback on the training provided via observations and a short online survey. Once all patient data were collected the qualitative researcher convened a focus group with the research nurses (one focus group was conducted) who were involved in patient recruitment and data collection. The research nurses who took part in the focus group were selected because they were directly involved in the recruitment and follow-up of patients. Telephone interviews were also conducted with physicians and nurses involved with the care of these patients. A total of nine semi structured interviews were conducted (3 with participants’ at one site and 2 from each of the remaining 3 sites). Details of interviewees are presented on Table 1.

**Table 1:** Details of research informants

Role	Label
Research Nurse	FGP1
Research Nurse	FGP2
Research Nurse	FGP3
Research Nurse	FGP4
Consultant in Emergency Medicine	Clinician 1
Consultant in Emergency Medicine	Clinician 2
Emergency Medicine Research Fellow	Clinician 3
Consultant in Emergency Medicine	Clinician 4
Advanced Clinical Practitioner (Nurse)	Clinician 5
Consultant in Emergency Medicine	Clinician 6
Consultant in Emergency Medicine	Clinician 7
Nurse Consultant	Clinician 8
Registrar	Clinician 9

Key: FGP Focus Group Participant

For the clinician interviews for convenience we initially approached Principal Investigators at sites who then used a process of snowballing to identify key people who the Principal Investigators felt had insights into the study [13]. We attempted to include clinicians who had not used the tool to gather insight on their perspectives. However, we were unable to find any clinicians who did not use the tool because they were unhappy with it. We did however identify and interview a clinician who had not used the tool because he had not seen an eligible patient during the intervention period.

Once all patient data were collected, the focus group was conducted in Birmingham. The focus group was used to explore the experiences of the research nurses in setting up and supporting the use of the intervention. The topic guide for the focus group included prompts about training received, identifying and consenting patients, ease of use, clinician response and data gathering.

The interviews were conducted over the phone. Each interview lasted around 30 min. A semi structured interview format was used, in which topics on the set up, use and observations regarding the PPT were explored. Participants were asked about their experience of prediction tools, their views of the STUMBL tool, their experience of their training in the use of the tool, their experience of using the tool and views on its potential role in the ED.

The focus group and interviews were audio recorded and these recordings were transcribed by a professional agency. The qualitative researcher checked the returned transcripts for accuracy and uploaded them into NVIVO 11 in order to complete the planned thematic analysis. All of the transcripts were checked by a second qualitative researcher using the coder comparison query tool in NVIVO11 with a pre-agreed target of 80% agreement between coders [14].

The qualitative data were coded and analysed using thematic analysis [15, 16]. The data presented in this paper are drawn from transcripts of one focus group with four research nurses and nine telephone interviews with clinicians.

## Results

Upon completion of the coder comparison query an average of 94.39% was achieved with only 5 areas of disagreement between the coders. These discrepancies were discussed and it was agreed that these discrepancies resulted from differences in coding style. A final percentage agreement of 94% was confirmed in discussions between the qualitative researcher and the second coder.

The qualitative researchers highlighted 21 themes through the process of constant comparison. These themes were identified as process themes (10); impact themes (8) and mainstreaming themes (3). Through a process of constant comparison these were further reduced to 9 themes: five process themes of study design; consent, collecting data; twitter and training were identified alongside two impact themes of making care decisions and reflective practice and finally two themes of looking to the future: mainstreaming and clinical need. Much of these data are presented in the results papers, which also considers possible improvements to the study design [17].

In this paper, we focus largely on the three themes we labelled under ‘impact’, including user friendliness, reflexivity, and guided action to explore the incorporation of the tool into clinical activity including the impact on patient care and the clinician approach to patients. These themes are highlighted here as they shed light on the ways in which the tool was used by clinicians to enable conversations about care planning and risk management. This heading covers the issues of shared decision-making and reflective practice and also highlights the ways in which the clinical teams used the tool to think through patient care and to arrange specialist services and ongoing care for the patients they saw. The clinicians’ highlighted instances where the tool changed the way they dealt with patients and with other healthcare professionals. Respondents also highlighted the role of the diagnostic tool in their discussions.

### Qualitative findings

#### User-friendliness

This theme covered the clinician’s experience of using the tool (data illustrating the themes are summarised in Table 2). This theme encompassed three main subthemes. These included the cognitive role of the tool, easing cognitive burdens, and patient centred communication.

**Table 2:** Major themes and sub-themes with illustrative quotes

Theme	Extract
**User friendliness**	
*Cognitive role of the PPT*	[…] and at the moment we try and figure out if they are going to be ok or not from their blunt rib injury, and I think it just draws together a number of factors that we would automatically think of that are really important. […] like how old are they and how frail are they maybe by looking at their lung disease or their age but it also brings into view other factors like anti-coagulation which I would not automatically have thought of. And also it seems to build into it a scoring system of how much weight we should attach to each of those variables that we are maybe juggling in an ethereal way in our head (Clinician4).
*Easing cognitive burdens*	I do think it reduced my workload because I did not, it sounds awful, but I did not have to think that much about the pre-existing problems with the patient because it was all a tick box for me basically (clinician5).
*Patient centred communication*	I think patients quite like if you use a scoring system. Because they can understand the risk prediction as well […] I have used it you know not just for this, but for other scoring systems that we use in ED, that as I said to patients “well you know I have put you through our scoring tool and you are high, medium or low risk”. I think patients might ask you about it, what is in it, and it is a good basis for conversation around shared decision making (clinician7)
**Reflexivity**	
*Reflecting on previous clinical decisions*	I think a few of the cases we scored greater than I would’ve expected, but that just sort of made me sort of step back and think perhaps in the past I maybe under treated some of this patient group. So it wasn’t a bad thing, it sort of helped me sort of reflect on my own practice (Clinician8)
	I had quite a few comments from the clinical staff being involved and were quite engaged in it, and it’s really been thought provoking for them […] by introducing the intervention they automatically start thinking about their clinical practice and they really sort of, they stand back a bit and go, “Ooh, I hadn’t thought about that.” So it does influence them (FGP1)
**Guiding action**	
*Alternative assessments*	literally just prompted him to take a step back and actually look at that particular patient in slightly more detail rather than just following a standard clinical process […] and it just added in an extra element that made him go, “Do you know what though, he has got a few risk factors I hadn’t really thought about and maybe I should have a look at him a bit more.” [FGP3]
*Smoothing referrals*	[…] with this tool you can refer to specialty and it’s something you can hang your hat on, and say, “I’ve done the blunt scoring tool, the score is 25.” And then they can also refer back to that and see, “oh yes, I can see where you’re scoring those points”. And it’s just something--, it just makes referral easier sometimes, asides from the fact that obviously if they come in as a less than ten, that’s considered discharge home. Obviously it says ‘consider’, […] But definitely having the tool, it kind of feels like everybody’s singing off the same hymn sheet (Clinician9)
*Actual resource requirements*	I think that discussion it created, it actually created more buy-in to the trial because it actually made them stop and think a bit. Because it did create quite a bit of discussion. (FGP1)
**Role of the diagnostic tool**	
*Observer status*	You throw in some sort of clarity in to how you have come to that decision and then what that means in the terms of percentages and outcomes. It [the STUMBL tool] can really just sharpen everyone’s thinking around it [prognosis] and they do not have to agree with you but at least you have got a basis rather than it just being an idea (Clinician4).
	I remember one patient I saw that I probably wouldn’t have considered in ITU but, you know, on their scoring and the injury that they had, I did have that conversation [about whether the patient needed to go to ITU]. So it doesn’t necessarily mean the patient is going to get admitted on to them [ITU], but--, under them [ITU staff], but they sort of open up that clinical discussion to ensure the patient’s got the right pathway and the right care plan (Clinician8)
*Validated tool*	I know myself and my ENP colleagues have sort of struggled with the fact that there is no sort of validated scoring tool for blunt chest wall trauma. So this was quite sort of an exciting step forward. And I know sort of in my role as teaching--, for teaching ENPs, some of the ENPs do struggle that there is no established tool compared with maybe other injuries (Clinician8).
	This is going to make my job easier. And it did because it gave me something that I could base my clinical decisions on (Clinician5).
*Questioning validation*	people were a bit, jumping the gun a bit, and saying well, like using this score, and saying I don’t know, this shows this, and I don’t know, there isn’t evidence yet (Clinican3).
	the real test is whether they actually go and use it properly in practice, for the rest of the patients. Or whether they just ignore it and do their own thing anyway. But with this decision it is actually really interesting that people wanted to follow it. They suggested to me that there was definitely a gap in the evidence people want to have an evidenced base tool to justify what they are doing for these patients and after the trial finished we had a number of people asking where we can get the decision rule from (Clinician2).

The tool drew together clinical assessments with additional observations that the clinicians felt were relevant and inspired confidence in the results of the tool. One clinician reflects on how the tool takes into account features of patient presentation that he “would automatically think of” together with additional factors which it combines in a scoring system that weights relevance of variables in a more systematic way than he would “in an ethereal way” (Clinician 4). As the tool inspired confidence, clinicians felt they could rely on this tool, and therefore it played a role in easing the cognitive burdens involved in assessing the potential severity of blunt chest wall trauma in light of pre-existing health conditions. Finally, some clinicians described how this PPT facilitated patient centred communication. In this regard, the PPT provided a kind of external point of view that was brought into the conversation between the clinician and the patient. That is to say, the clinician can explain to a patient that they have used a scoring system, and the outcome of this system. This facilitates a conversation between the clinician and the patient on the tool and its indicators. In this way, the tool provides the clinician with a way of communicating a prognosis without having to associate themselves with the prognosis.

#### Reflexivity

A second theme that emerged coalesced around the opportunity the tool created for clinicians to take a step back from their decisions to think and consider how they have managed these patients. The status of the tool as an external viewpoint provided clinicians with a framework with which to evaluate their past clinical decisions, allowing clinicians to consider how they would have assessed these patients compared with how the PPT guided their actions. In some ways, the introduction of a prognostic tool into an ED serves as a prompt to communication and reflection among staff. This was something commented on by research nurses. To a large extent, the newness of the tool and the timing of its introduction into an ED will itself have an effect on the team, and bring about some degree of change to clinical practice. However, what the reflexivity theme highlights is how the tool illuminates the importance of unexpected prognostic factors for the condition. The awareness of additional prognostic indicators highlighted by the tool may initiate sustained changes in practice for some clinicians, or initiate a long term commitment to using PPTs.

#### Guiding action

The PPT was designed to guide decisions about treatment and the management of relevant patients. Guiding action became an important theme both for clinicians and for the nurses involved. Within this theme, subthemes emerged on alternative assessments, smoothing referrals, and identifying resource requirements.

The guiding action theme emerged in different ways. One of the research nurses described how a clinician told her he didn’t think a patient qualified for the trial and was about to discharge a patient. Feeling uncertain of this decision she approached and consented the patient for the trial, completing the PPT. This illustrates how the presence of the prediction tool in the clinic provided a research nurse with a mechanism to produce an alternative assessment and prompt additional clinical inquiries. Clinicians also described using the tool to gain access to other specialties within the hospital and to get buy-in to their care plan from other clinical teams. In such cases, the PPT provided a transparent and comprehensible framework that could be applied to cases allowing clinicians to use the tool’s prognostic scores to smooth referrals. This had a knock-on effect on workloads in other departments who experienced “an increase in workload which they weren’t particularly happy with, but they have come around to it now” (Clinician 5). Finally, the guiding action theme also emerged in descriptions of how the tool provided an opportunity for clinicians and managers to reflect on the practices and resources that are realistically needed to manage the conditions experienced by this expanding population. This led to conversations on the actual resources required to manage this patient group.

#### Added element - the role of the diagnostic tool

As shown above, over the course of the interviews it became clear that the usefulness of the prediction tool was based on its capacity to guide prognosis. The capacity of the PPT to guide stemmed, in part, from its quasi social role in the ED. The PPT is both a form of knowledge (condensed into a form) and is a guide, implying it occupies a social role. This social role gave rise to subthemes on the observer status of the tool in the ED, and questions regarding its validation.

The social role theme was manifested in the status of the PPT as a knowledge based assessment. As such, the PPT occupied a social location amongst practitioners but was also external to the social group. In a sense, the PPT took on this social role as its score and guidance could be understood as implicitly commenting on clinical decisions. In examples of this theme the PPT acts like a neutral external observer insofar as the evidence basis of the prediction and the overall score become an utterance that acting clinicians feel they need to take into account in making decisions. Thus, the tool is itself a quasi-social actor in the ED providing clarity on a decision in relation to an individual patient and prompting conversations on patient management. In both these cases, the tool effects clinician communication allowing conversations to shift as the tool ensures the conversation has “got a basis” (Clinician 4) so that all participants are able to understand the situation in the same way.

The role of the tool was in part based on its scientific validation. Here, clinicians focus on the cognitive form of the tool, emphasising the importance of ascertaining the reliability and validity of the tool as a prediction model. The validated nature of the tool was not without question, with at least one clinician querying certain elements. Nevertheless, the knowledge contained in the tool itself and its potential to aid clinical assessments in complex cases appeared to be justified and motivates the STUMBL team to pursue a full effectiveness trial to facilitate its widespread use.

## Discussion

This qualitative analysis highlights the ways in which good prognostic models provide clinicians with tools that aid communication with other clinicians and with patients. By concentrating on the effect of prediction models to assist professionals in their decision making, and to aid clinicians communicate with patients about their decisions, we can identify how PPTs help to better manage patients. This should in turn lead to unlocking the final element of the Baker and Gerdin model: improved outcomes [10].

The data presented here adds qualitative depth to the arguments proffered by Baker and Gerdin [10]. As they show, research on the use of PPTs have tended to focus on the predictive performance of the tools and on their effects on patient outcomes. They submit that the implementation and adoption of these tools are based on additional features of the tool and how the tool may fit into clinical practice. In this paper, we use qualitative data gathered on the incorporation of a single PPT into EDs to add empirical insight to these features. We show that the success of the PPT under consideration here stems in part from the how it could be incorporated into clinical practice. The user friendly nature of the tool and its use of clinically comprehensible indicators inspired confidence and facilitated patient/professional communication. The PPT prompted reflexive awareness in clinicians, by promoting reflection on past clinical decisions and highlighting the importance of additional prognostic factors. The PPT also provides a guide to clinical decision making. The guiding theme relates to the scope for the PPT to expand the capacity of nurses to influence prognostic outcomes, and for clinicians to use the PPT score to facilitate the referral of patients. Finally, the PPT takes on a social role in the ED. As a knowledge based assessment, the PPT score can play a role as an external commentator on clinical thinking, and provides common ground for discussions among clinicians about patient outcomes. This suggests that while this PPT provided an aid for clinicians in prognosticate outcomes for their patients, it also changed clinical practice by aiding communication and rebalancing hierarchies of power and influence within the ED.

### Limitations

The analysis presented here is based on a small qualitative research package accompanying a feasibility trial. The qualitative work was set up to explore attitudes to the study and reasons for compliance or non-compliance with the protocol. It was not focused on the use or implementation of PPTs in general, but on the clinician experience of incorporating one specific prediction tool into their clinical activities as part of a feasibility trial. The core of the analysis is based on telephone interviews with a small convenience sample of clinicians working in EDs at four sites. Attention must therefore be drawn to the small number, and the possibility that these clinicians may have had particularly favourable attitudes to the PPT. They were all at sites that had agreed to participate in the trial and this will also have had an effect on their willingness to prognosticate. Nevertheless, the fact that many of the participants in this research are senior clinicians with a strong background in research may mitigate the risk of unsubstantiated bias. Research that focuses explicitly on the implementation and role of PPTs in EDs is needed to fully explore the themes identified here.

## Conclusion

This paper examines the intuition that PPTs have complex effects on clinical practice, and maps some of these effects. Our research adds empirical weight to Baker and Gerdin [10] by showing that the usefulness of a PPT like the one examined here, stem as much from its usefulness in clinical practice in the ED as from its performance in predicting outcomes for patients. Because this paper is based on the experience of incorporating a single PPT into EDs as part of a feasibility trial, its findings are non-generalizable. However, we conclude by arguing that there is a need to explore not only how well PPTs perform in the ED, but how PPTs are incorporated into clinical practice and how they impact culture and practice among nurses and clinicians in EDs.

## Data Availability

The identifiable data that support the findings of this study are not publicly available. Restrictions apply to the availability of the non-identifiable data.

## Abbreviations

PPT: Prognostic Prediction Tool
ED: Emergency department
STUMBL: STUdy evaluating the impact of a prognostic model for Management of BLunt chest wall trauma patients
FGP: Focus Group Participant
